# The Alpha Isoform of Heat Shock Protein 90 and the Co-chaperones p23 and Cdc37 Promote Opioid Anti-nociception in the Brain

**DOI:** 10.3389/fnmol.2019.00294

**Published:** 2019-11-29

**Authors:** Wei Lei, David I. Duron, Carrie Stine, Sanket Mishra, Brian S. J. Blagg, John M. Streicher

**Affiliations:** ^1^Department of Pharmacology, College of Medicine, University of Arizona, Tucson, AZ, United States; ^2^Department of Pharmaceutical and Administrative Sciences, School of Pharmacy, Presbyterian College, Clinton, SC, United States; ^3^Department of Chemistry & Biochemistry, College of Science, University of Notre Dame, Notre Dame, IN, United States

**Keywords:** heat shock protein 90 (Hsp90), p23, Cdc37, opioid, pain, anti-nociception, CRISPR, translation

## Abstract

Opioid activation of the mu opioid receptor (MOR) promotes signaling cascades that evoke both analgesic responses to pain and side effects like addiction and dependence. Manipulation of these cascades, such as by biased agonism, has great promise to improve opioid therapy. However, the signaling cascades of the MOR are in general poorly understood, providing few targets for drug development. In our earlier work, we identified Heat shock protein 90 (Hsp90) as a novel and crucial regulator of opioid anti-nociception in the brain by promoting ERK MAPK activation. In this study, we sought to identify the molecular isoforms and co-chaperones by which Hsp90 carried out this role, which could provide specific targets for future clinical intervention. We used novel selective small molecule inhibitors as well as CRISPR/Cas9 gene editing constructs delivered by the intracerebroventricular (*icv*) route to the brains of adult CD-1 mice to target Hsp90 isoforms (Hsp90α/β, Grp94) and co-chaperones (p23, Cdc37, Aha1). We found that inhibition of the isoform Hsp90α fully blocked morphine anti-nociception in a model of post-surgical paw incision pain, while blocking ERK and JNK MAPK activation, suggesting Hsp90α as the main regulator of opioid response in the brain. We further found that inhibition of the co-chaperones p23 and Cdc37 blocked morphine anti-nociception, suggesting that these co-chaperones assist Hsp90α in promoting opioid anti-nociception. Lastly, we used cycloheximide treatment in the brain to demonstrate that rapid protein translation within 30 min of opioid treatment is required for Hsp90 regulation of opioid response. Together these studies provide insight into the molecular mechanisms by which Hsp90 promotes opioid anti-nociception. These findings thus both improve our basic science knowledge of MOR signal transduction and could provide future targets for clinical intervention to improve opioid therapy.

## Introduction

The mu opioid receptor (MOR) evokes complex signal transduction cascades upon activation by opioid ligands like morphine. For decades now it has been understood that the MOR represses cAMP production by inhibiting adenylyl cyclase *via* the Gα_I_ subunit, and activates G protein-coupled inwardly-rectifying potassium channels *via* the Gβ/γ subunit, which summate on neuronal hyperpolarization and subsequent inhibition of nociceptive inputs (Al-Hasani and Bruchas, 2011). However, it is clear that signaling regulators beyond this simple cascade have a strong impact on opioid anti-nociception and side effects, including other G proteins, ERK MAPK (Macey et al., 2009), Src (Zhang et al., 2017), CaMKII (Li et al., 2016), RSK2 (Darcq et al., 2012), and others. These signaling regulators could provide important targets for opioid drug development; for instance βarrestin2 was shown to reduce opioid anti-nociception while promoting side effects like tolerance and dependence, leading to the development of βarrestin2 biased agonists with reduced side effects (Bohn et al., 1999; Raehal et al., 2005; Dewire et al., 2013; Manglik et al., 2016; Schmid et al., 2017). However, in general, the mechanisms by which these signaling regulators impact opioid physiology are not known, and very few targets like βarrestin2 have been validated for drug development (Al-Hasani and Bruchas, 2011; Olson et al., 2017). This gap illustrates the need for investigation into the signalosome of the MOR and the mechanisms by which these regulators impact opioid physiology.

To this end, in our earlier work, we identified the central signaling regulator Heat shock protein 90 (Hsp90) as a novel and crucial regulator of opioid signaling in the brain, that promoted opioid anti-nociception by promoting ERK MAPK activation (Lei et al., 2017). Hsp90 is a major regulator of protein folding *via* chaperone activity in concert with other Hsps like Hsp70 (Li and Buchner, 2013). However, Hsp90 also has a major role in signal transduction by regulating signaling molecule localization, complex/scaffold formation, and acute signaling activation (Streicher, 2019). Despite the importance of Hsp90 in regulating signaling, only two previous studies *directly* linked Hsp90 to opioid signaling. An *in vitro* study found that Hsp90 inhibition decreased cAMP superactivation, a marker for opioid dependence (Koshimizu et al., 2010); supporting these findings, an *in vivo* mouse study found that injection of Hsp90 inhibitor reduced the somatic signs of morphine withdrawal (Abul-Husn et al., 2011). Our study was thus the first to *directly* link Hsp90 regulation of MOR signaling to opioid anti-nociception.

Our study did show that Hsp90 inhibition very strongly decreased morphine anti-nociception in models of acute and chronic pain, and identified a signaling mechanism *via* ERK MAPK (Lei et al., 2017). However, this study only took the first small step in identifying the role of Hsp90 in regulating opioid signaling. We used the ATP-pocket inhibitor 17-AAG, which is non-selective between the four Hsp90 isoforms (Hsp90α/β, Grp94, TRAP1). These isoforms differ in their subcellular localization and protein targets, with Hsp90α/β localized to the cytoplasm, Grp94 to the endoplasmic reticulum, and TRAP1 to the mitochondria (Liu et al., 2015; Kim et al., 2016; Mishra et al., 2017). We also did not identify any of the crucial co-chaperones, which mediate and target the specific activity of Hsp90 in different cells and tissues (Li and Buchner, 2013). Co-chaperones have specific roles, like Cdc37 having a key role in signaling kinase targeting, suggesting their possible involvement in MOR signaling (Hinz et al., 2007). Identifying the isoforms and co-chaperones involved in Hsp90 regulation of opioid signaling will thus reveal key details of the molecular mechanism by which Hsp90 promotes anti-nociception. Identifying these refined molecular targets could also provide more selective targets for clinical intervention, which has been done in an analogous way for Hsp70 (Assimon et al., 2013, 2015).

In this study, we thus sought to identify specific Hsp90 isoforms and co-chaperones responsible for the promotion of opioid anti-nociception by Hsp90. We utilized novel selective inhibitors and *in vivo* CRISPR/Cas9 gene editing in the brains of adult CD-1 mice to test Hsp90 isoforms (Hsp90α/β, Grp94) and co-chaperones (p23, Cdc37, Aha1). Through these studies, we found that the isoform Hsp90α and the co-chaperones p23 and Cdc37 strongly promoted MOR signaling and opioid anti-nociception in the brain. These findings expand our knowledge of the specific molecular mechanisms by which Hsp90 regulates opioid anti-nociception, and could provide more selective targets for clinical intervention.

## Materials and Methods

### Drugs

KUNA115 (Mishra et al., under review), KUNB106 (Mishra et al., in press), KUNG65 (compound 30 in Crowley et al., 2017), KU-32 (compound A4 in Ansar et al., 2007), and KU177 (compound 12c in Zhao et al., 2011) were synthesized by the Blagg laboratory using the cited protocols. The identity of the ligands was confirmed by high resolution mass spectrometry and nuclear magnetic resonance, while the purity of the compounds was confirmed to >95% by high performance liquid chromatography. Gedunin (#33-871-0), Celastrol (#32-031-0), 17-AAG (#AAJ66960MC), and Cycloheximide (#AC357420010) were obtained from Fisher Scientific. DAMGO (#1171) was obtained from Tocris/R&D. Morphine sulfate pentahydrate was obtained from the NIDA Drug Supply Program. All compounds except for DAMGO and morphine were prepared as DMSO stock solutions and diluted into a vehicle solution prior to injection. DAMGO was prepared in a stock solution of sterile USP water and morphine in sterile USP saline; morphine was prepared fresh prior to each experiment. Matched vehicle controls were included for each drug injection. The vehicles used were: 2% DMSO and 98% sterile USP water for KUNA115, KUNB106, KUNG65, KU177, Cycloheximide; 1% DMSO and 99% sterile USP water for KU-32 and 17-AAG; 10% DMSO, 10% Tween80, and 80% sterile USP water for gedunin and celastrol; sterile USP water for DAMGO; and sterile USP saline for morphine. Drug powders were stored at −20°C under desiccation or as recommended by the manufacturer, and stock solutions were stored at −20°C.

### CRISPR/Cas9 DNA Constructs

CRISPR gene editing constructs were obtained from Genecopoeia as all-in-one DNA vectors containing universal promoters driving expression of the gRNA and Cas9 gene, along with a neomycin resistance gene for mammalian cell selection and an mCherry gene for visualization (pCRISPR-CG vector). The constructs were pre-designed by Genecopoeia to target each mouse gene. They included a universal negative control vector that expresses all the same elements with a non-targeting gRNA (#CCPCTR01-CG01-B), and constructs to target Hsp90α (#MCP229411-CG01), p23 (#MCP232080-CG12), Cdc37 (#MCP231406-CG12), and PEBP1 (#MCP231756-CG01) as a further negative control.

Each DNA vector was amplified for use using standard bacterial transformation, and an endotoxin-free maxi-prep kit to reduce inflammation upon injection. Each vector was also validated by restriction digest. The Hsp90α vector was validated in an *in vitro* experiment. Mouse 66.1 breast cancer cells were cultured as described in Edwards et al. (2018). The cells were electroporated with 10 μg of DNA per cuvette, then selected with 500 μg/ml of G418 until the cells recovered and began growing again. At this point, the cells were harvested and analyzed by Western blot as described below. The *in vivo* delivery and validation of all vectors are also described below.

### Animals

Male and female CD-1 (a.k.a. ICR) mice from 4 to 8 weeks of age were used for all experiments and were obtained from Charles River Laboratories. The mice were recovered for at least 5 days after shipping prior to use and housed no more than five per cage. All mice were housed in the University of Arizona’s AAALAC-accredited vivarium with temperature and humidity control, 12 h light/dark cycles, and standard chow and water available *ad libitum*. All experiments performed were approved by the University of Arizona’s IACUC, and all experiments were in accordance with the NIH Care and Use of Laboratory Animals handbook.

### Paw Incision Model

Mice were randomly assigned to experimental groups in age-matched cohorts, and the experimenter was blinded to treatment group identity by the delivery of coded drug vials. We utilized a post-surgical paw incision pain model, with the surgery performed as described in our earlier work (Lei et al., 2017). Drugs or CRISPR DNA constructs were delivered by the intracerebroventricular (*icv*) route in a 5 μl volume, also performed as described in Lei et al. (2017). For drug treatments, the paw incision surgery was performed, and while the mice were still under anesthesia, an *icv* injection of inhibitor drug or vehicle was performed. The mice then recovered from both the surgery and injection for 1 or 24 h prior to opioid injection and pain measurement. For CRISPR experiments, 4 μg of DNA was complexed with Turbofect *in vivo* transfection reagent (#FERR0541 from Fisher Scientific) according to the manufacturer’s instructions and injected *icv* daily from days 1 to 3. The mice then recovered, with the paw incision surgery performed on day 9 and opioid injection and pain measurement performed on day 10. Our *in vivo* CRISPR protocol is based on the protocol reported in Sandweiss et al. (2017).

Mechanical pain/allodynia on the incised paw was measured using Von Frey filaments with the up-down method, as performed in our earlier work and the literature (Chaplan et al., 1994; Lei et al., 2017; Edwards et al., 2018). Pre- and post-CRISPR and pre- and post-surgical baselines were measured to determine any impact of the treatment on baseline responses prior to the injection of morphine. Mechanical allodynia was measured in a 2–3 h time course after the injection of morphine.

### Signaling Protein Analysis by Western Blot

To analyze brain signaling changes, mice were injected *icv* with KUNA115 as above for 24 h, followed by *icv* injection of DAMGO for 10 min. The mice were sacrificed by rapid cervical dislocation, and the periaqueductal gray (PAG) region was rapidly dissected on an ice-cooled metal block and snap-frozen in liquid nitrogen. G418-selected populations of 66.1 cells transfected with CRISPR constructs described above were also harvested for protein analysis. The cells were washed with ice-cold dPBS and the cells recovered by adding lysis buffer and scraping with a plastic cell spatula. The methods for protein extraction from both brain regions and cell lysates, the composition of our lysis buffer, and the protocol for performing the Western blot are all reported in our earlier work (Lei et al., 2017).

We used the following antibodies for our Western analysis: phospho-Akt (#50-191-224, Fisher Scientific); total-Akt (#50-190-279, Fisher Scientific); phospho-ERK (#50-191-932, Fisher Scientific); total-ERK (#50-191-129, Fisher Scientific); phospho-JNK (#9255, Cell Signaling); total-JNK (#9252, Cell Signaling); Hsp70 (#4872, Cell Signaling); STAT3 (#9139, Cell Signaling); GAPDH (#PIMA515738, Fisher Scientific); Hsp90α (#MA110892, Fisher Scientific); and mCherry (#NBP196752SS, Fisher Scientific). The antibodies were generally used at 1:1,000 in 5% BSA in TBST rocking overnight at 4°C. We used goat anti-rabbit or goat anti-mouse IRDye 680 or 800 secondary antibodies from LiCor Biosciences at 1:5,000–10,000 in 5% non-fat dry milk in TBST at room temperature for 1 h. The resulting data was imaged using an Odyssey Fc imager from LiCor Biosciences. The data were quantitated using Scion Image, derived from NIH ImageJ. Phospho-protein signal was normalized to total protein signal from the same sample (e.g., pERK normalized to tERK), while total protein signal was normalized to the housekeeping gene GAPDH (e.g., STAT3 normalized to GAPDH). These normalized data were further normalized to the vehicle-treated control animals within each experiment.

### Immunohistochemistry

CRISPR-mediated knockdown in the brain of the target proteins Hsp90α, p23, and Cdc37 was validated *post hoc* in treated mice as above using immunohistochemistry. The mice were first perfused with 4% paraformaldehyde in saline, and the brains removed and frozen as a block in OTC medium. The brains were sectioned using a cryotome with 20 μm sections and mounted on Leica Xtra slides (#NC0215141, Fisher Scientific); the frozen sections were dried at room temperature for 15 min and stored at −20°C until use.

For Hsp90α: sections warmed to room temperature, then washed in TBS (20 mM Tris, 150 mM NaCl, pH 7.2) for 10 min. Blocked for 2 h in a humidified chamber at room temperature (10% goat serum, 0.3% Triton-X100 in TBS). Incubated with primary antibody (#380-003, Synaptic Systems) at 1:50 in 5% goat serum, 0.3% Triton-X100 in TBS overnight at 4°C. Washed 3 × 10 min in TBS, followed by anti-rabbit Alexa 488 secondary antibody (#A11034, Fisher Scientific) at 1:200 in 5% goat serum, 0.3% Triton-X100 in TBS for 1 h at room temperature. The slides were then washed 3 × 10 min in TBS, dried at room temperature for 10 min, then mounted.

For p23 and Cdc37: sections warmed to room temperature, then washed in phosphate buffered saline (PBS) for 10 min. Heat-induced antigen retrieval performed for 20 min at 95°C in sodium citrate buffer (10 mM sodium citrate, 0.05% Tween20, pH 6.0). The slides were cooled to room temperature for 20 min, then washed in PBS with 0.1% Tween20 (PBST) twice, and PBS once. The sections were then blocked for 30 min in a humidified chamber at room temperature [3% fetal bovine serum (FBS) in PBS]. After blocking, the sections were incubated with primary antibody overnight at 4°C (p23-1:1,000 of #MA3414 from Fisher Scientific; Cdc37-1:100 of #MA3029 from Fisher Scientific; both in 3% FBS in PBS). The slides were then washed 3 × 10 min in PBST, followed by secondary antibody at room temperature (p23-1:200 of anti-mouse Alexa594, #A11032 from Fisher Scientific, in 3% FBS in PBS for 1 h. Cdc37-1:200 of anti-mouse Alexa488, #A11031 from Fisher Scientific, in 3% FBS in PBST for 30 min). The slides were then washed 3 × 10 min in PBST and dried at room temperature for 10 min before mounting.

All sections were imaged using a standard fluorescent microscope using the appropriate filters for Alexa488 (blue/green) and Alexa594 (green/red). The knockdown of all targets was broadly apparent across the brain. The pontine reticular nucleus (PRN) was chosen as the site of imaging and quantitation. Images were taken from 4 to 6 adjacent sections from each animal. The fluorescence intensity divided by the area of the image was calculated for each section, and the 4–6 sections per animal averaged to produce a single mean value counted as *N* = 1.

### Data Analysis

All data is reported as the mean ± SEM. All statistical analysis was performed using GraphPad Prism 8.0. All behavioral data is reported as raw threshold values without normalization; Western blot and immunohistochemistry data normalized as described in those sections above. Statistical comparisons for behavioral data and ERK/JNK/Akt Western data performed using a two-way ANOVA with Fisher’s Least Significant Difference *post hoc* test. Comparisons of immunohistochemistry data and Hsp70/STAT3 Western data performed using an Unpaired 2-Tailed *t*-test. In all cases significance was set as a *p*-value of <0.05. For the dose/response experiment using KUNA115, the area under the curve (AUC) was calculated for each dose and treatment using Prism 8.0, and graphed by log dose and treatment group. Linear regression analysis was performed, and the parameters of those fitted lines used to calculate the potency (A_50_) as previously described (Lei et al., 2017). The sample sizes and technical replicates for each experiment are described in the Figure Legends.

## Results

### Isoform-Selective Inhibitor Screen Identifies Hsp90α

**Figure 1:**
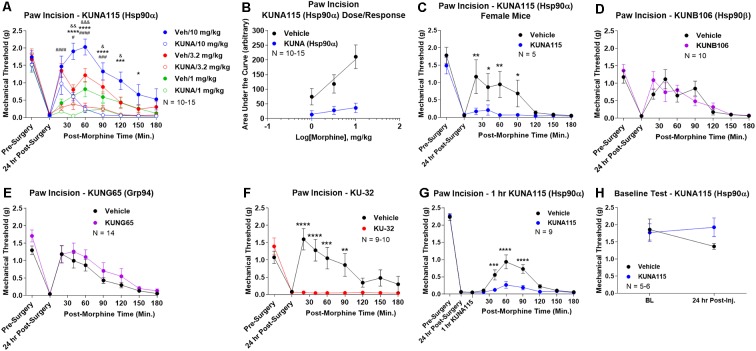
Heat shock protein 90 (Hsp90) isoform-selective inhibitor screen identifies Hsp90α in promoting opioid anti-nociception in the brain. CD-1 mice had the paw incision surgery performed, with concurrent injection of vehicle or 0.1 nmol of inhibitor intracerebroventricular (*icv*). Mice recovered 24 h, followed by *sc* injection of morphine. Mechanical thresholds measured before and after surgery, and in a time course after morphine injection. Pre- and post-surgery baselines did not differ for any group (*p* > 0.05). All data reported as the mean ± SEM, with sample sizes of mice/group noted in each graph. All statistics performed by two-way ANOVA with Fisher’s Least Significant Difference *post hoc* test. **(A)** Male mice tested with KUNA115 (Hsp90α selective) and a 1–10 mg/kg morphine dose range. *,***,****p < 0.05, 0.001, 0.0001 between Veh and KUNA groups at same time point at 10 mg/kg; ^#,###,####^*p* < 0.05, 0.001, 0.0001 between Veh and KUNA groups at same time point at 3.2 mg/kg; ^&,&&,&&&^*p* < 0.05, 0.01, 0.001 between Veh and KUNA groups at same time point at 1 mg/kg. Performed in 2–3 technical replicates. **(B)** Dose/response curves constructed from the data in **(A)** and analyzed as described in “Materials and Methods” section. A_50_: Vehicle = 8.95 mg/kg; KUNA115 = 88, 139, 382 mg/kg (too flat for accurate calculation). **(C)** KUNA115 experiment performed in female mice with 3.2 mg/kg morphine. Performed in one technical replicate. For remaining graphs: *,**,***,****p < 0.05, 0.01, 0.001, 0.0001 vs. same time point inhibitor treatment group. **(D)** Male mice tested with KUNB106 (Hsp90β selective) with 3.2 mg/kg morphine. Performed in two technical replicates. **(E)** Male mice tested with KUNG65 (Grp94 selective) with 3.2 mg/kg morphine. Performed in three technical replicates. **(F)** Male mice tested with KU-32 (non-selective) with 3.2 mg/kg morphine. Performed in two technical replicates. **(G)** Male and female mice had paw incision performed, with 23 h recovery. KUNA115 or Vehicle then injected as above, with 1 h treatment prior to 3.2 mg/kg morphine. Performed in two technical replicates. **(H)** Male and female mice had KUNA115 or Vehicle injected as above with a 24 h recovery. Pre- and post-injection baselines measured without any surgery, pain state, or opioids present. Performed in two technical replicates by different experimenters.

To identify the active Hsp90 isoform in regulating opioid anti-nociception in the brain, we performed a screen of isoform-selective small molecule inhibitors. KUNA115 is selective for Hsp90α (Mishra et al., under review), KUNB106 for Hsp90β (Mishra et al., in press), and KUNG65 for Grp94 (Crowley et al., 2017). All inhibitors were delivered at a screening dose of 0.1 nmol by the *icv* route for 24 h, a model predicated on our earlier studies with the non-selective Hsp90 inhibitor 17-AAG (Lei et al., 2017). Over a full morphine dose range of 1–10 mg/kg, we found that KUNA115 strongly blocked morphine anti-nociception in paw incision pain, suggesting the involvement of the isoform Hsp90α (Figure 1A). Dose/response analysis for this experiment revealed an A_50_ potency value of morphine of 8.95 mg/kg for Vehicle-treated mice, in line with literature values, validating the experiment (Figure 1B). The dose/response curve for KUNA115 meanwhile was so flat it did not give a feasible value (calculated A_50_ of 88, 139, 382 mg/kg; Figure 1B). These results show that an Hsp90α-selective inhibitor strongly blocked morphine anti-nociception in this pain model in line with our previous results using a non-selective inhibitor (Lei et al., 2017). We also found a similar result with female mice, suggesting no sex differences with this target and model (Figure 1C).

When we tested the other isoform-selective inhibitors, we found no differences for KUNB106 (Hsp90β; Figure 1D) and KUNG65 (Grp94; Figure 1E). These results do suggest that Hsp90α alone is active in the brain for opioid signaling regulation. As a further control, we tested the impact of an alternate site inhibitor KU-32, which binds to the C-terminal region of Hsp90 unlike the ATP pocket targeted 17-AAG but is similarly non-selective between isoforms (Ansar et al., 2007). The results with KU-32 are the same as for KUNA115 above and 17-AAG (Lei et al., 2017), further validating the results and suggesting a *bona fide* role for Hsp90 in regulating opioid anti-nociception (Figure 1F).

We also performed additional experiments to further define the impact of KUNA115/Hsp90α on opioid anti-nociception. All experiments above were carried out with a 24 h recovery, leaving the time course of KUNA115 unknown. We thus performed a paw incision experiment as above with only a 1 h KUNA115 treatment; this resulted in a strong loss of morphine anti-nociception, similar to the 24 h results above (Figure 1G). This finding suggests that KUNA115 has a relatively rapid onset that is sustained for 24 h or more. We also controlled for potential impacts of KUNA115 on mechanical thresholds without pain or opioids present. We found that a 24 h KUNA115 treatment as above had no impact on baseline mechanical thresholds, suggesting the results above are due to a specific impact on the opioid system (Figure 1H). This conclusion is further supported by our earlier work in which we found no impact of Hsp90 inhibitor treatment on motor performance in the Rotarod test (Lei et al., 2017).

### Hsp90α Regulates Opioid Signal Transduction

**Figure 2:**
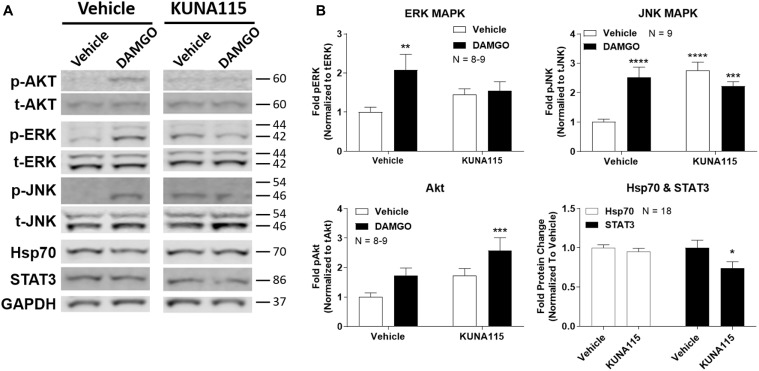
Hsp90α regulates opioid signal transduction in the brain. Male CD-1 mice had vehicle or 0.1 nmol KUNA115 injected *icv*, 24 h, followed by vehicle or 0.1 nmol DAMGO *icv*, 10 min. Periaqueductal gray region analyzed by Western blot. All data reported as the mean ± SEM, with sample sizes of mice/group noted in each graph; all experiments performed in four technical replicates. For ERK, JNK and Akt, data analyzed by two-way ANOVA with Fisher’s Least Significant Difference *post hoc* test; **,***,****p < 0.01, 0.001, 0.0001 vs. Vehicle:Vehicle group. For Hsp70 and STAT3 data analyzed by Unpaired 2-Tailed *t*-test; *p < 0.05 vs. same target Vehicle group. **(A)** Representative sample blots shown for each target, with MW indicated for each protein. Each pair of images for one target (e.g., p-Akt) were from the same blot, but discontinuous, so they are separated to denote this fact. **(B)** All Western data quantitated by target. ERK, JNK, and Akt are phosphorylated protein signal normalized to total protein. Hsp70 and STAT3 are normalized to GAPDH. KUNA115 treatment caused a loss of ERK and JNK stimulation over baseline by DAMGO, and a loss of STAT3 protein expression.

We next sought to measure the impact of Hsp90α-selective inhibition on opioid signaling. We combined KUNA115 treatment with DAMGO stimulation in the brain, which is a highly selective, potent, and efficacious MOR agonist. We analyzed ERK and JNK MAPK, Akt, Hsp70, and STAT3 by Western blot in the PAG region of the brain (Figure 2A). This region was chosen based on our earlier studies using non-selective inhibitors (Lei et al., 2017); the PAG is also a key region in the pain modulatory circuitry (Heinricher et al., 2009). We found that both ERK and JNK MAPK phosphorylation was stimulated by DAMGO in Vehicle-treated mice, however, stimulation over baseline was lost with KUNA115 treatment (Figure 2B). With JNK MAPK, KUNA115 significantly raised the unstimulated baseline, as we saw for ERK MAPK with 17-AAG treatment (Lei et al., 2017). With the kinase Akt, KUNA115 treatment tended to increase both unstimulated and DAMGO-stimulated phospho-Akt levels, so that the KUNA115/DAMGO group was significantly elevated over Vehicle/Vehicle baseline (Figure 2B). Lastly, KUNA115 treatment had no impact on Hsp70 protein levels, unlike 17-AAG treatment (Lei et al., 2017), while it significantly decreased total protein levels of the signaling regulator STAT3 (Figure 2B). Together these results demonstrate that Hsp90α has a specific role in regulating opioid signal transduction in the brain suggesting potential involvement in opioid anti-nociception, similar to what we showed for 17-AAG treatment and ERK MAPK (Lei et al., 2017).

### CRISPR Knockdown of Hsp90α in Adult Mouse Brain

**Figure 3:**
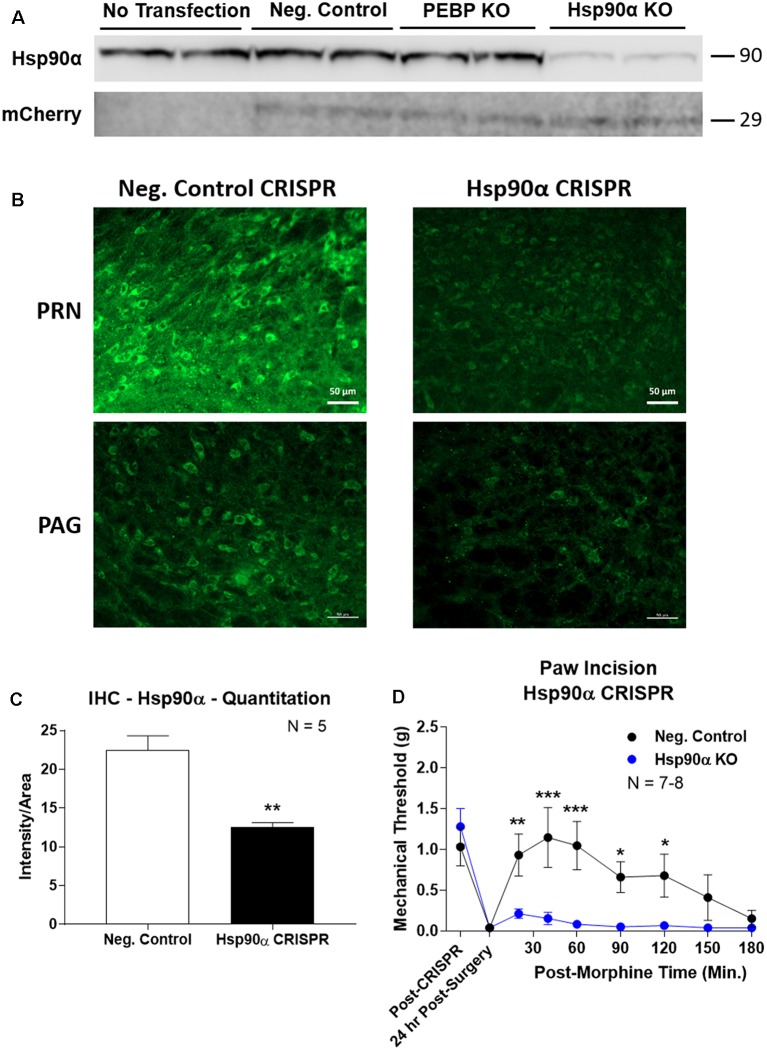
CRISPR/Cas9 gene editing of Hsp90α in adult mouse brain. CRISPR constructs for all targets prepared and delivered as described in “Materials and Methods” section. All quantitative data reported as mean ± SEM. **(A)** Hsp90α CRISPR construct validated in 66.1 cells. Western blot shown with replicate wells of cells in each lane, with MW indicated for each target. Hsp90α protein levels reduced by ~90% only in the presence of Hsp90α-targeted CRISPR construct. Other constructs (Negative Control, PEBP) have no effect. mCherry protein levels are present in all transfected cells, demonstrating successful transfection of CRISPR DNA. **(B)** Hsp90α or negative control CRISPR delivered to CD-1 male mouse brains and analyzed for protein knockdown on day 10. Representative images shown from pontine reticular nucleus (PRN) and periaqueductal gray (PAG). Hsp90α (green signal) is present in cell bodies and dendritic trees, and the signal is reduced by CRISPR treatment. **(C)** Quantitation of all data from **(B)** performed as described in “Materials and Methods” section. Sample size of mice/group noted in graph. **p < 0.01 vs. Negative Control group by Unpaired 2-Tailed *t*-test. Mice treated in one technical replicate, with the resulting tissue stained and analyzed in more than one technical replicate. CRISPR treatment reduced Hsp90α signal by 43.9%. **(D)** CRISPR-treated CD-1 male mice had paw incision surgery performed on day 9, with injection of 3.2 mg/kg morphine *sc* on day 10. Sample size of mice/group noted in graph, performed in two technical replicates. *,**,***p < 0.05, 0.01, 0.001 vs. same time point Hsp90α group by two-way ANOVA with Fisher’s Least Significant Difference *post hoc* test.

To confirm the role of Hsp90α in regulating opioid anti-nociception, we used CRISPR/Cas9 DNA constructs to knockdown Hsp90α protein expression broadly across the brain in adult mice (similar approach to Sandweiss et al., 2017). We first validated our CRISPR construct *in vitro* using mouse 66.1 cells. Transfection of a negative control CRISPR vector or a vector targeting the protein PEBP had no impact on Hsp90α protein levels, while our vector targeting Hsp90α reduced protein levels by ~90% (Figure 3A). We could also detect expression of the mCherry protein in all cells with a CRISPR vector, verifying successful transfection of all constructs (Figure 3A).

We next treated mice with the Hsp90α or negative control CRISPR vectors as described in the “Materials and Methods” section, and validated successful target knockdown by immunohistochemistry. We found broad knockdown across the entire brain and selected the PRN and PAG for analysis. We could detect a strong signal in both cell bodies and apparent dendritic fields that was strongly reduced by Hsp90α CRISPR treatment (Figure 3B). Quantitation of fluorescent signal revealed a significant reduction of 43.9% (Figure 3C). Thus validated, we next tested the impact of Hsp90α knockdown on morphine anti-nociception (Figure 3D). We found that Hsp90α CRISPR treatment fully blocked anti-nociception in paw incision pain (Figure 3D), very similarly to KUNA115 (Figure 1) and 17-AAG (Lei et al., 2017), confirming the role of Hsp90α in regulating opioid anti-nociception in the brain.

### Hsp90 Co-chaperones p23 and Cdc37 Regulate Opioid Anti-nociception in the Brain

**Figure 4:**
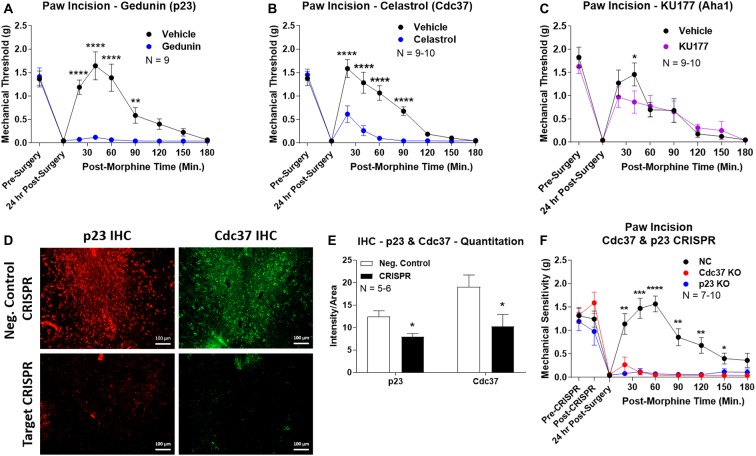
Identification of the co-chaperones p23 and Cdc37 as promoters of opioid anti-nociception in the brain. Male CD-1 mice used for all experiments, data reported as the mean ± SEM, with sample sizes of mice/group noted in each graph. **(A–C)** Ten nanomoles of Gedunin (p23, **A**), 10 nmol of Celastrol (Cdc37, **B**), or 0.1 nmol of KU177 (Aha1, **C**) or Vehicle control injected *icv* concurrently with paw incision surgery with a 24 h recovery, followed by 3.2 mg/kg morphine *sc*. Experiments performed with two technical replicates for each drug. *,**,****p < 0.05, 0.01, 0.0001 vs. same time point inhibitor treatment group by two-way ANOVA with Fisher’s Least Significant Difference *post hoc* test. **(D)** p23, Cdc37, or Negative Control CRISPR-treated mice with *icv* delivery of constructs analyzed by IHC for target knockdown on day 10. Representative images shown from the PRN. Both targets (p23 – red, Cdc37 – green) have a similar staining pattern to Hsp90α, and both are clearly reduced by CRISPR treatment. **(E)** Data from **(D)** for all mice quantitated as described in the “Materials and Methods” section. All mice treated in one technical replicate, with staining and analysis of the resulting tissue performed in more than one technical replicate. *p < 0.05 vs. same target Negative Control group by Unpaired 2-Tailed *t*-test. CRISPR treatment reduced p23 signal by 36.3% and Cdc37 by 46.0%. **(F)** CRISPR-treated mice had paw incision surgery performed on day 9, with injection of 3.2 mg/kg morphine *sc* on day 10. Performed in two technical replicates. *,**,***,****p < 0.05, 0.01, 0.001, 0.0001 vs. both same time point p23/Cdc37 CRISPR groups by two-way ANOVA with Fisher’s Least Significant Difference *post hoc* test.

We first used the co-chaperone-selective inhibitors gedunin (p23, Brandt et al., 2008), celastrol (Cdc37, Zhang et al., 2008), and KU177 (Aha1, Zhao et al., 2011) in paw incision pain as for the isoform inhibitors above. Both gedunin (p23; Figure 4A) and celastrol (Cdc37; Figure 4B) strongly reduced morphine anti-nociception in paw incision pain, very similar to the Hsp90 inhibitors above. However, KU177 (Aha1) had only a slight impact on opioid anti-nociception, suggesting it may not have a significant role (Figure 4C).

We next moved forward with CRISPR/Cas9 gene editing to confirm that p23 and Cdc37 regulate opioid anti-nociception as we did for Hsp90α above. IHC analysis showed a similar broad knockdown across the brain with CRISPR treatment, particularly apparent in the PRN (Figure 4D). Quantitation revealed significant decreases of 36.3% for p23 and 46.0% for Cdc37 (Figure 4E). We next tested the impact of targeted CRISPR treatment for these proteins in paw incision pain, and found that both p23 and Cdc37 CRISPR knockdown fully blocked morphine anti-nociception, very similar to Hsp90α inhibition above or 17-AAG treatment (Lei et al., 2017; Figure 4F). These results confirm that both p23 and Cdc37 regulate opioid anti-nociception in the brain.

### Rapid Translation Required for Hsp90 Regulation of Opioid Anti-nociception

**Figure 5:**
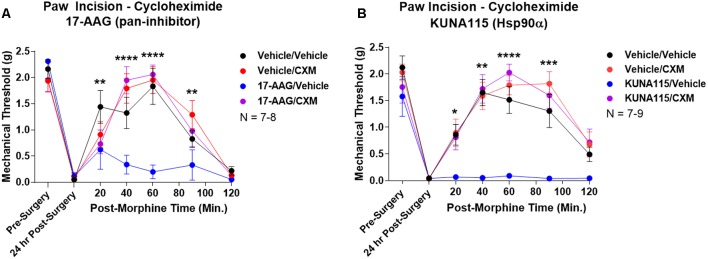
Rapid protein translation is required for Hsp90 inhibition to impact opioid anti-nociception. Male and female CD-1 mice treated with 0.5 nmol 17-AAG **(A)** or 0.1 nmol KUNA115 **(B)** or vehicle *icv*, 24 h, followed by 85 nmol cycloheximide or vehicle *icv*, 30 min, followed by 10 mg/kg morphine *sc*. No difference between approximately equal male and female groups (*p* > 0.05) so they were combined for this analysis. Data reported as the mean ± SEM with the sample size of mice/group noted in the graph. Performed in three technical replicates by different experimenters. *,**,***,****p < 0.05, 0.01, 0.001, 0.0001 for the 17-AAG/Vehicle group vs. any of the other three groups at the same time point by two-way ANOVA with Fisher’s Least Significant Difference *post hoc* test. There were no differences between the Vehicle/Vehicle, Vehicle/CXM, or 17-AAG/CXM groups.

Lastly, we sought to identify part of the molecular biology mechanism by which Hsp90 regulates opioid anti-nociception. We combined 24 h Hsp90 inhibition as above and in our previous work (Lei et al., 2017) with treatment of the translation inhibitor cycloheximide in the brain 30 min prior to morphine treatment. We found that cycloheximide had no impact on morphine anti-nociception in vehicle-treated mice; however, cycloheximide fully restored morphine anti-nociception back to vehicle-treated levels in mice treated with the pan-Hsp90 inhibitor 17-AAG (Figure 5A). We found the same results with KUNA115, confirming that Hsp90α regulates translation during morphine anti-nociception (Figure 5B). These results strongly suggest that rapid translation within 30 min of morphine treatment is required for Hsp90 inhibition to impact opioid signaling and anti-nociception.

## Discussion

**Figure 6:**
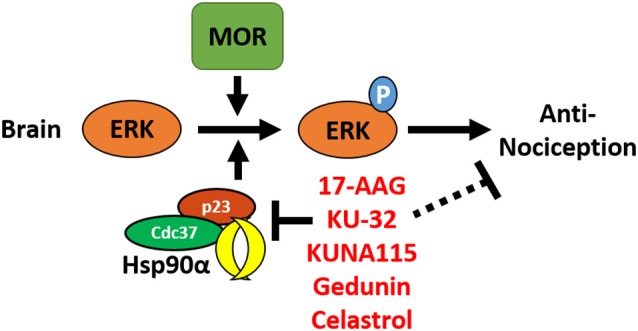
Model for the regulation of mu opioid receptor (MOR) signaling by Hsp90 in the brain. This summary model combines results from this manuscript with our earlier work using non-selective Hsp90 inhibitors in the brain (Lei et al., 2017). Together this data suggests that Hsp90α, p23, and Cdc37 act in concert in the brain to promote the phosphorylation of ERK MAPK by the MOR in response to opioid drugs, thus promoting opioid anti-nociception. Treatment with non-selective or selective inhibitors blocks this role, thus blocking ERK MAPK phosphorylation and blocking anti-nociception in response to opioids.

In this study, we have identified the isoform Hsp90α as a key player in promoting opioid anti-nociception and signaling in the brain using both selective small molecule inhibitors (Figures 1, 2) and CRISPR/Cas9 gene editing in the brains of adult mice (Figure 3). Using these techniques, we further identified the co-chaperones p23 and Cdc37 as key promoters of opioid anti-nociception (Figure 4). We also found that rapid protein translation is part of the molecular mechanism by which Hsp90 regulates opioid anti-nociception (Figure 5). When combined with the results of our previous study using a non-selective Hsp90 inhibitor (Lei et al., 2017), our findings suggest that Hsp90α in concert with p23 and Cdc37 promote ERK MAPK activation by the MOR in the brain, and that inhibiting these proteins reverses these roles summating in loss of opioid ERK activation and anti-nociception. This model is diagrammed in Figure 6.

Understanding the specific molecules involved in promoting opioid anti-nociception may provide future targets for refined clinical intervention. First generation non-selective Hsp90 inhibitors like 17-AAG failed clinical trials due to liver toxicity (Sidera and Patsavoudi, 2014). Second generation and alternate site inhibitors like KU-32 have shown higher tolerability, and indeed KU-32 has been shown to be neuroprotective (Urban et al., 2010, 2012; Ma et al., 2015). However, non-selective targeting of Hsp90 has an inherently higher risk of unacceptable side effects, especially since Hsp90 is a ubiquitous protein with a very high expression level and numerous client proteins (Li and Buchner, 2013). The four Hsp90 isoforms have different cellular locations (cytoplasm for Hsp90α/β, endoplasmic reticulum for Grp94, mitochondria for TRAP1) and client proteins, meaning that targeting only one necessary isoform, such as Hsp90α identified above, should inherently decrease the risk of side effects (Echeverría et al., 2011). Similarly, identifying specific co-chaperones like p23 and Cdc37 identified above will also provide specific targets that should reduce potential side effects. This is particularly true with co-chaperones since there are many more of them and each co-chaperone is more selective by role and tissue expression, providing further selectivity (Li and Buchner, 2013). Along these lines, recent studies have identified ligands that interfere with specific Hsp90:co-chaperone interactions, such as celastrol used in our study here, that impact specific functions without the broad sledgehammer of inhibiting the entire Hsp90 protein (Zhang et al., 2008). The specific proteins identified through this study could thus be the first step in identifying improved therapies to modulate opioid treatment; an analogous approach has been used for Hsp70 (Assimon et al., 2013).

The identification of these specific molecules may also lend insight into the molecular mechanism by which Hsp90 regulates opioid signaling. As mentioned above, Hsp90α is cytoplasmic with its own unique complement of client proteins (Bergmayr et al., 2013; Liu et al., 2015). This specificity narrows down the field of potential mechanisms by which Hsp90α regulates opioid signaling, especially to the Hsp90α-specific signaling changes in ERK, JNK, and STAT3 that we have identified (Figure 2). Further differences include the lack of Hsp70 induction caused by KUNA115 treatment in Figure 2. Earlier studies have shown that non-selective inhibitors like 17-AAG impact both the heat shock response (Hsp70 induction) and protein folding activity of Hsp90, while the C-terminal inhibitor KU-32 only impacts the heat shock response (Ansar et al., 2007). All three compounds, 17-AAG, KU-32, and KUNA115 (Figure 1; Lei et al., 2017), impact opioid anti-nociception in the same way, providing a clue that the protein activity of Hsp90α responsible will be the one impacted by all three classes of compound (and which does not require Hsp70 upregulation).

Similarly, the co-chaperone Cdc37 that we have identified has been shown in numerous studies to be crucial for targeting kinases to Hsp90 in both canonical and non-canonical pathways that are crucial for kinase function (Hinz et al., 2007; Gould et al., 2009; Ota et al., 2010). Importantly, these studies have shown that Cdc37 does not simply assist in kinase folding and maturation, but also assists in complex formation that is required for acute activation. Considering the signaling kinases impacted, especially ERK MAPK which we have identified as a mechanism of Hsp90 regulation of anti-nociception (Figure 2; Lei et al., 2017), it makes sense that Cdc37 would be implicated in the Hsp90 mechanism of action. The co-chaperone p23 also has a canonical role in assisting Hsp90 in the protein folding cascade, and has also been linked to acute regulation of signal transduction, as by the A2A receptor (Bergmayr et al., 2013; Li and Buchner, 2013). The functional overlap between the specific molecules identified and the drugs used will allow us to identify likely mechanisms of signaling regulation in future studies, further informed by our results in Figure 5 suggesting that active protein translation is required for Hsp90 inhibition to impact opioid anti-nociception. Our results showing that Hsp90 inhibition is impactful within 1 h and that baseline mechanical response is not altered provides further mechanistic guidance (Figures 1G,H).

While positively identifying Hsp90α, p23, and Cdc37, our studies demonstrated no response to other isoform and co-chaperone inhibitors. This does suggest that Hsp90β, Grp94, and Aha1 are not involved in regulating opioid anti-nociception in the brain. However, this data must be interpreted with caution. We did not exhaustively test these molecules by CRISPR and other methods as we did for Hsp90α, p23, and Cdc37, leaving open the possibility that these other molecules could still be involved. Thus our data should be interpreted most strongly as identifying Hsp90α, p23, and Cdc37 without necessarily ruling out other players. Future studies can address this question more exhaustively, as well as test for the involvement of numerous other co-chaperones not tested in this study. These studies were also all performed broadly across the forebrain without testing the impact of the spinal cord or the periphery, or sub-regions and circuits within the brain. Future studies will also need to address whether these other regions have different mechanisms by which Hsp90 could regulate opioid response.

## Data Availability Statement

All datasets generated for this study are included in the article.

## Ethics Statement

The animal study was reviewed and approved by IACUC, University of Arizona.

## Author Contributions

WL collaboratively developed the initial idea for the project, participated in study design, performed most experiments, and analyzed the data. DD performed some paw incision and cycloheximide experiments, and analyzed the data. CS performed one of the cycloheximide experiments and analyzed the data. SM synthesized, purified, and characterized the novel small molecule inhibitors described above. BB supervised SM in the course of the chemistry work, and collaboratively developed the initial idea for the project. JS supervised WL, DD, and CS in the course of their work, conceived the initial idea for the project, participated in study design, analyzed some of the data, and wrote the manuscript. All authors had editorial input into the manuscript.

## Conflict of Interest

JS has grants from the NIH and Arizona Biomedical Research Commission (ABRC), as well as research contracts with Depomed, Inc. and Divine Healing, LLC. JS also is a founder with an equity stake in Teleport Pharmaceuticals, LLC. None of these commercial involvements concern the topic of the research study at hand, or Hsp90 research more broadly. BB has grants from the NIH and is a founder with an equity stake in Grannus Therapeutics, a virtual startup for developing novel Hsp90 inhibitors.

The remaining authors declare that the research was conducted in the absence of any commercial or financial relationships that could be construed as a potential conflict of interest.
